# Massive brainstem infarction and high-risk pulmonary embolism successfully treated with pharmaco-invasive approach: A case report

**DOI:** 10.1097/MD.0000000000042434

**Published:** 2025-05-16

**Authors:** Qiong-Fang Yang, Cai-Min Shu

**Affiliations:** a Department of Respiratory Medicine, Affiliated Dongyang Hospital of Wenzhou Medical University, Dongyang, Zhejiang, China.

**Keywords:** brainstem infarction, case report, high-risk, pulmonary artery thrombectomy, pulmonary embolism

## Abstract

**Rationale:**

Concurrent acute massive brainstem infarction and high-risk pulmonary embolism (PE) present a critical therapeutic dilemma due to contraindications for thrombolysis in acute stroke. Such cases are rarely reported, and optimal management strategies remain undefined. This case highlights the challenges and underscores the importance of timely mechanical intervention in life-threatening dual pathologies.

**Patient concerns:**

A 63-year-old man was admitted to the hospital with partial paralysis of his left leg, which had been unresponsive for 7 hours. In the emergency department, magnetic resonance imaging of the head showed multiple recent infarctions of the brainstem and right cerebellar hemisphere. The patient was therefore diagnosed with an acute cerebral infarction. After nearly a week of treatment, including anticoagulation and plaque stabilization, his condition improved significantly. On his seventh day of hospitalization, after waking up and going to the bathroom, his oxygenation suddenly decreased, accompanied by hypotension and shock.

**Diagnoses:**

After a comprehensive analysis, we considered the possibility of a PE. Subsequent computed tomographic pulmonary angiography confirmed this to be the case, pointing to a massive high-risk lesion.

**Interventions:**

Anticoagulation with unfractionated heparin failed to stabilize hemodynamics. Multidisciplinary consensus prioritized pulmonary artery thrombectomy with catheter-directed thrombolysis, avoiding systemic thrombolysis risks.

**Outcomes:**

Post-thrombectomy, hemodynamic stability was restored. Anticoagulation was successfully transitioned from unfractionated heparin to rivaroxaban, achieving complete thrombus resolution at 3 months. The patient regained functional capacity without hemorrhagic complications.

**Lessons:**

This case demonstrates that pulmonary artery thrombectomy is a viable lifesaving option for high-risk PE when thrombolysis is contraindicated in acute stroke. It emphasizes the role of multidisciplinary decision-making and mechanical interventions in dual critical pathologies, offering a framework for managing similar complex cases.

## 1. Introduction

Acute infarction of the brainstem, which occurs mainly in elderly patients, has high rates of incidence, disability, and mortality; pulmonary embolism (PE) is among its serious complications and is one of the main causes of death following acute cerebral infarction.^[1,2]^ The peak incidence of PE is mainly in the second to 4th weeks after cerebral infarction, partly owing to stroke, which leads to a limitation of activity.^[3]^ In the acute phase of stroke, once PE has developed, anticoagulation therapy is contraindicated because it would increase the risk of cerebral infarction bleeding conversion.^[4]^ In patients with high-risk PE, thrombolysis is contraindicated during the acute phase of cerebral infarction, making the choice of treatment more challenging. To the best of our knowledge, there are as yet no clinical reports on acute large-area brainstem infarctions complicated by high-risk PE. Here, therefore, we report such a case in order to improve clinicians’ understanding of similar situations.

## 2. Case presentation

**Figure 1.**
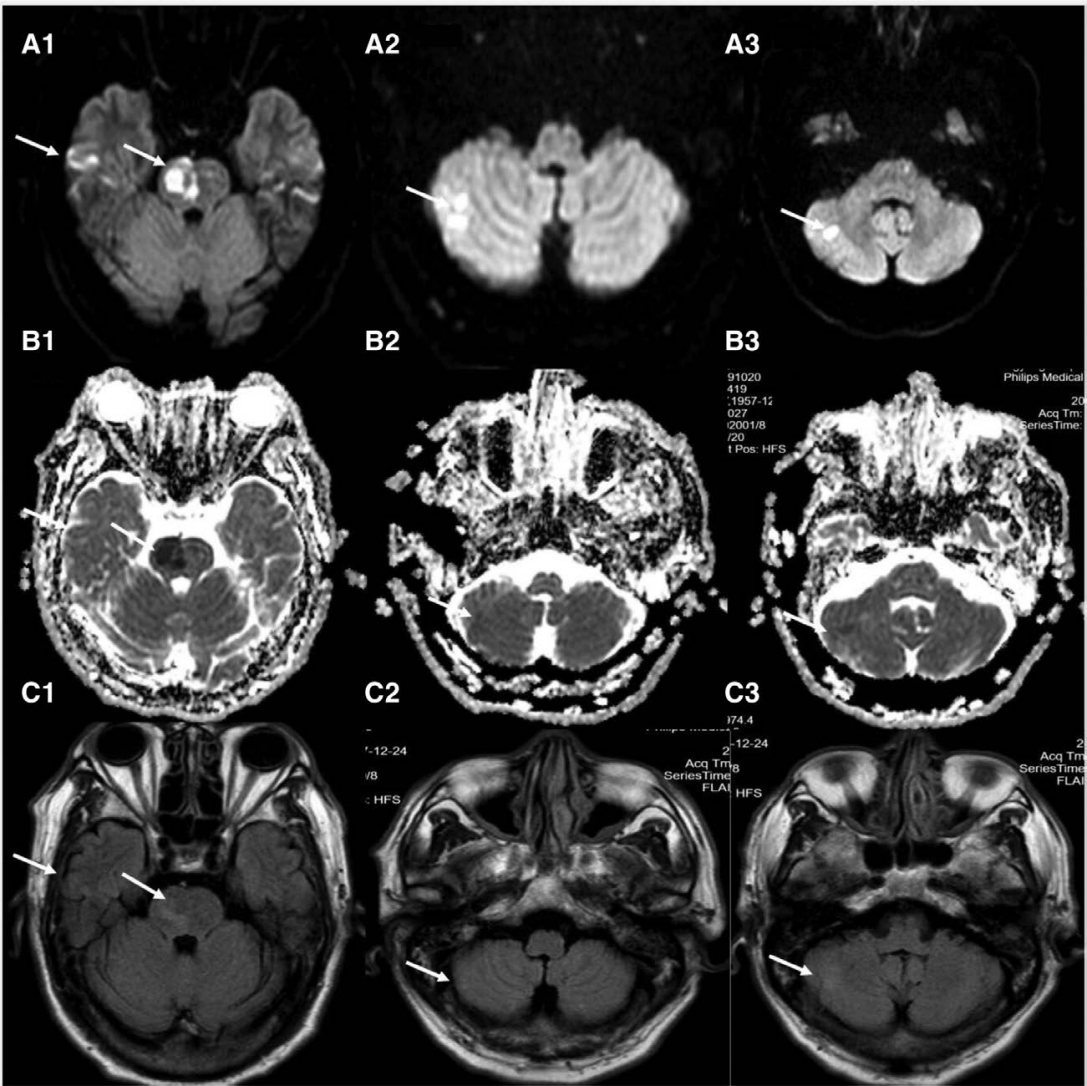
On admission, magnetic resonance imaging of the head showed recent multiple infarcts in the brainstem and right hemisphere. (A1–A3) Represent the DWI sequence. (B1–B3) Represent the apparent diffusion coefficient sequence, and (C1–C3) represent the T2 Flair sequence. The white arrows indicate the locations of the infarcts.

A 63-year-old man was admitted to our hospital on August 11, 2021, with a main complaint of partial paralysis, saying that his left leg had become unresponsive. He had a history of hypertension, which had been controlled with nifedipine. He had also smoked some 20 cigarettes/day for more than 30 years. Upon admission to the hospital, he reported having experienced 7 hours of weakness in his left leg, and he was unable to get up. Moreover, his responses were slow and his voice was unclear. His score on the Glasgow Coma Scale was 14, and his NIH Strike Scale and Rankin scores were 8 and 4, respectively. He was therefore sent to the emergency department, where he immediately underwent computed tomography (CT) and computed tomography angiography of the head and neck. The CT scan showed no obvious cerebral hemorrhage, while the computed tomography angiography suggested irregular thickening and calcification of the wall at the bifurcation of the right common carotid artery, with mild stenosis of the lumen. Also noted were calcified plaques in the siphon area of the internal carotid arteries, with mild narrowing of the lumen; the left vertebral artery was also slender. These findings indicated that the acute cerebral infarction should be considered first. Magnetic resonance imaging of the head revealed multiple recent infarcts in the brainstem and right cerebral hemisphere (Fig. 1). The patient was therefore given enteric-coated aspirin tablets (100 mg) and clopidogrel bisulfate (150 mg) to oppose platelet aggregation and atorvastatin (40 mg) to stabilize the plaque. He was then transferred to the neurology ward, where urinary kallidinogenase and edaravone were administered intravenously for almost a week. As a result, the patient condition improved significantly; his left leg gained muscle strength and his speech gradually returned to near normal. During hospitalization, this patient had a padua score of 4, suggesting a high risk of venous thrombosis, but because of the high risk of anticoagulant bleeding, a plantar vein pump was given for physical prophylaxis of venous thrombosis.

**Figure 2.**
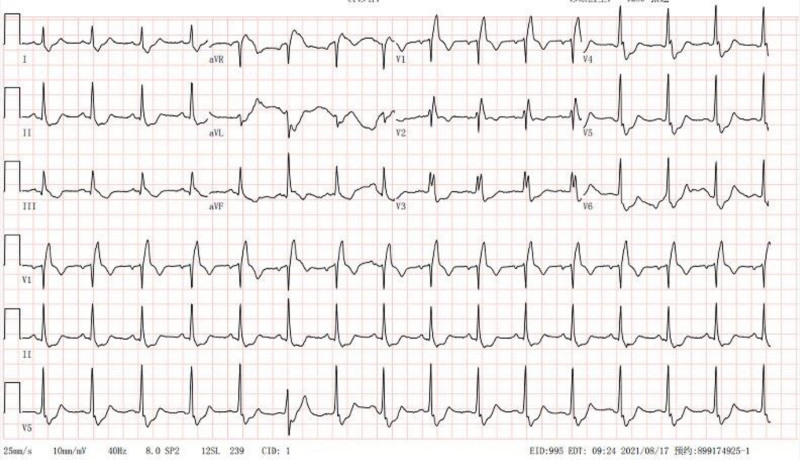
One week after acute cerebral infarction, bedside electrocardiography showed typical SIQIIITIII changes.

**Figure 3.**
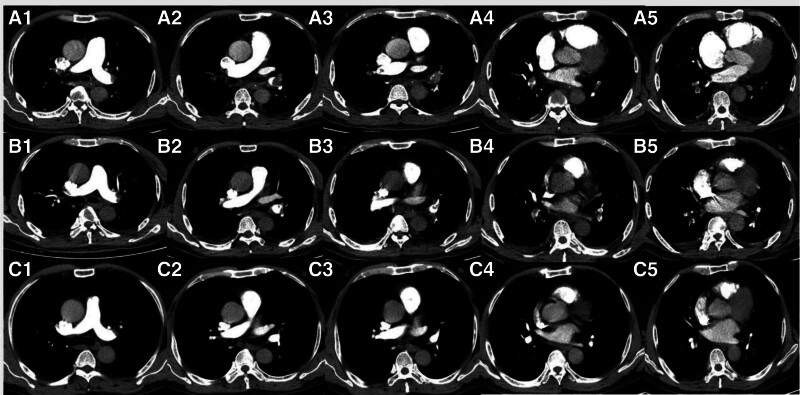
(A1–A5) One week after acute cerebral infarction, computed tomographic pulmonary angiography showed the left and right pulmonary artery trunks and grades II–III major branch emboli. (B1–B5) Five days after pulmonary artery thrombectomy, CTPA showed emboli in the trunks and main branches of the left and right pulmonary arteries. These were significantly absorbed compared with the initial stage of the disease. (C1–C5) Three months after discharge, a CTPA reexamination showed that there are no emboli in the main pulmonary arteries or their branches. CTPA = computed tomography pulmonary angiography.

**Figure 4.**
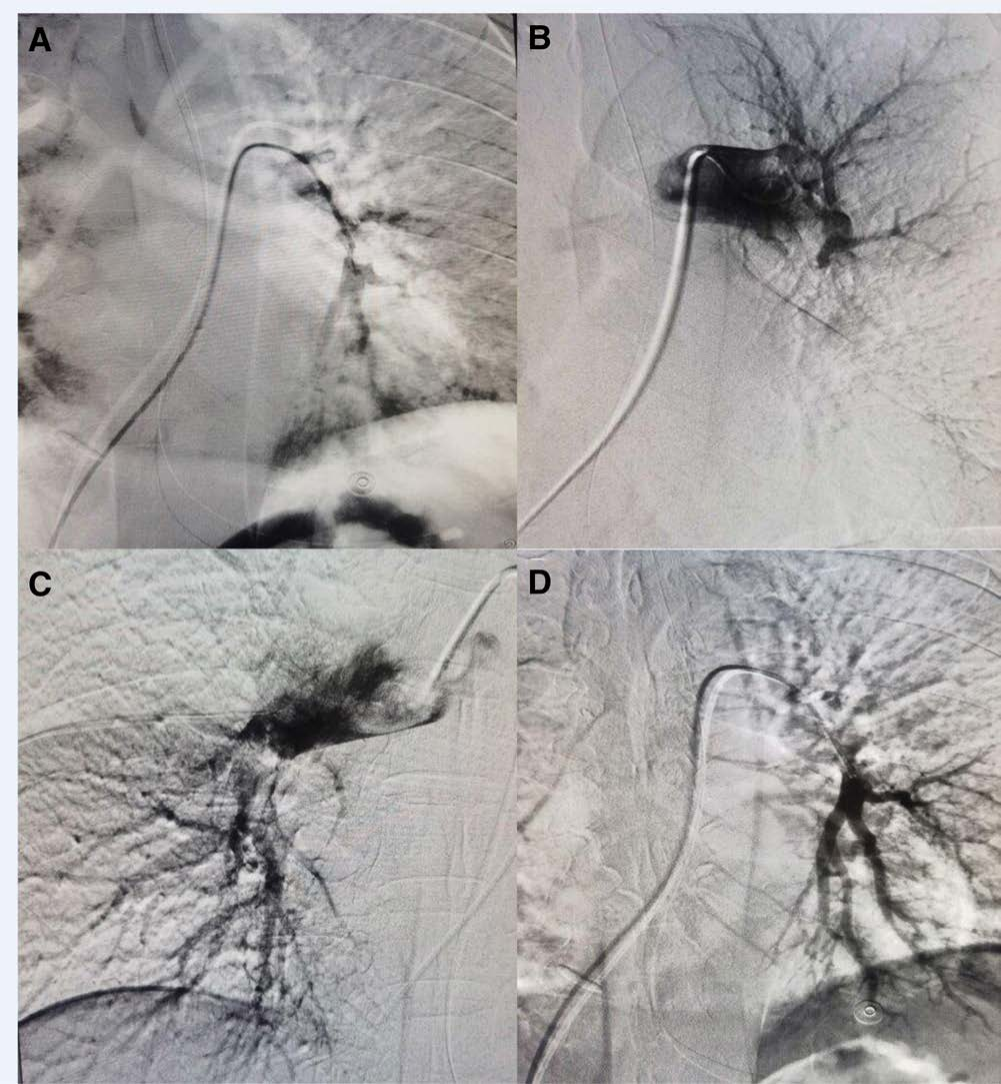
(A–C) Pulmonary angiography during surgery showed multiple thromboses in the main left and right pulmonary arteries and their branches. (D) Postoperative pulmonary angiography showed that the left pulmonary artery and its branches were unobstructed, with no obvious thrombosis.

On the seventh day post admission, after getting up to go to the toilet, the patient experienced sudden hypotensive shock with decreased oxygenation. Physical examination: BP 72/52 mm Hg; P, 96 beats/min; R, 24 beats/min; SO_2_, 88%; clear consciousness. Clear breath sounds in both lungs, with no dry or moist rales. A regular heart rhythm, soft abdomen, no edema in the limbs, and with negative Babinski sign on both sides. As a result, the patient immediately received treatment with a high-flow oxygen mask to enhance oxygenation and an intravenous injection of norepinephrine (1.6 mg/h) to maintain blood pressure. However, his condition did not improve. Laboratory examination: arterial blood gas analysis showed that when the oxygen concentration was 33%, the blood oxygen partial pressure was 62.7 mm Hg, the carbon dioxide partial pressure was 31.9 mm Hg, the oxygenation index was 190 mm Hg, and the blood lactate was 1.5 mmol/L, indicating type I respiratory failure; the levels of white blood cells, C-reactive protein, and calcitonin were basically normal. Blood chemistry examination indicated only hypercalcemia, while other indicators were within the normal range. The level of serum D-dimer increased significantly, to 14.76 µg/mL, while the normal range was <0.5 µg/mL. The levels of plasma pro-brain natriuretic peptide and troponin-T were 243.7 pg/mL and 0.016 ng/mL, respectively (Table 1). Bedside electrocardiography showed typical SIQIIITIII changes (Fig. 2). Color Doppler ultrasound of the heart showed aortic sclerosis. The ejection fraction was 76%. Color Doppler ultrasound of blood vessels in both lower limbs revealed thrombosis of the left posterior tibial vein. Considering the patient bedridden state after cerebral infarction, acute onset of shock after getting out of bed, and characteristic changes on electrocardiography, the presence of a high-risk PE became the first consideration. Therefore, emergency computed tomographic pulmonary angiography (CTPA) was immediately performed. It showed that the left and right pulmonary artery trunks were obstructed and that there was a grade II–III PE in a major branch (Fig. 3A1–5). Ultimately, the diagnosis of high-risk PE was clear. However, the patient was in the acute stage of a massive brainstem infarction, and thrombolysis was contraindicated. Therefore, anticoagulation therapy was given with unfractionated heparin (first dose, 4000 IU IV; maintenance dose, 900 IU/h; followed by micropump maintenance). However, the blood pressure could not be stabilized, even with anticoagulation. After a joint consultation with experts from cardiac surgery, interventional department, and respiratory department, it was decided to perform pulmonary artery thrombectomy. Pulmonary angiography during the operation showed multiple thromboses in the main pulmonary artery and branches of the left and right pulmonary arteries (Fig. 4A–C). We used a 6F catheter to enter the left and right pulmonary artery trunks and branches and repeatedly pushed and pulled the catheter to break the thrombus; a large portion of thrombus was then extracted by manual thrombus aspiration. reexamination of pulmonary angiography revealed that the left and right pulmonary arteries and their main branches were unobstructed, and no obvious thrombosis was found (Fig. 4D). Postoperatively, urokinase (200,000 U) was given for selective transcatheter thrombolysis by the neurologist. After the procedure, the pulmonary artery pressure was measured at 45/28 mm Hg. The patient was then transferred to the intensive care unit (ICU). After the norepinephrine was stopped, his blood pressure returned to normal and his vital signs remained stable.

**Table 1:** Laboratory indicators at the onset of pulmonary embolism.

Test	Results	Reference range
White blood cell count	6.96 (10^9^/ L)	3.5–9.5
Neutrophil ratio	61.4 (%)	40–75
Lymphocyte ratio	24.9 (%)	20–50
Monocyte ratio	11.1 (%)	3–10
C-reactive protein (CRP)	16.15 (mg/L)	<5
Procalcitonin	0.036 (ng/mL)	<0.1
Serum sodium	140.2 (mmol/L)	137–147
Serum chlorine	106.7 (mmol/L)	99–110
Serum potassium	4.44 (mmol/L)	3.5–5.3
Serum calcium	2.69 (mmol/L)	2.02–2.6
Serum aspartate aminotransferase (AST)	22 (U/L)	15–40
Serum alanine aminotransferase (ALT)	28 (U/L)	9–50
Creatine kinase (CK)	42 (U/L)	50–310
Creatine kinase isoenzyme (CK-MB)	23 (U/L)	≤25
Serum lactate dehydrogenase (LDH)	148 (U/L)	120–250
D-dimer	14.76 (ug/mL)	<0.5
Fibrinogen (FIB)	4.17 (g/L)	2–4
Pro-B-type natriuretic peptide	243.7 (pg/mL)	5–125
Troponin-T	0.016 (ng/mL)	<0.1
Arterial partial pressure of oxygen (PaO_2_)	62.7 (mm Hg)	80–100
Arterial carbon dioxide pressure (PaCO_2_)	31.9 (mm Hg)	35–45
Oxygenation index (PaO_2_/FiO_2_)	190 (mm Hg)	400–500

ALT = serum alanine aminotransferase, AST = serum aspartate aminotransferase, CK = creatine kinase, CK-MB = creatine kinase isoenzyme, CRP = C-reactive protein, FIB = Fibrinogen, LDH = serum lactate dehydrogenase.

In the ICU, maintenance anticoagulation with unfractionated heparin was continued for 2 days. Because the patient condition was now stable, he was transferred to the medical ward. There, the anticoagulant regimen was changed to nadroparin calcium injection (4100 IU, I.H., q12h). Two days later, nadroparin calcium was dosed (6150 IU, I.H., q12h), during which occult blood was noted in the stool along with a slight abnormality in the liver enzymes, which improved after symptomatic treatment. After anticoagulation for 5 days postoperatively, CTPA reexamination showed emboli in the trunks and main branches of the left and right pulmonary arteries, which were significantly absorbed compared with the initial stage of the disease (Fig. 3B1–5). Because the patient left-leg muscle strength had decreased after cerebral infarction, he was subsequently transferred to the rehabilitation department for rehabilitation training.

**Figure 5.**
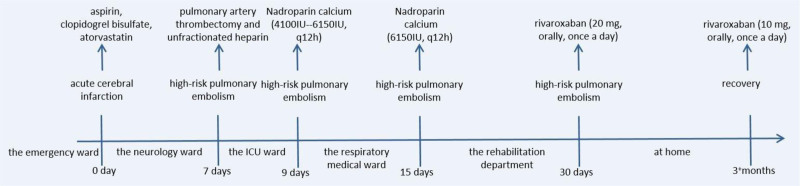
Timeline of the patient’s hospitalization and treatment.

During his hospitalization in the rehabilitation department, the patient anticoagulant treatment with nadroparin calcium continued. On being discharged from the rehabilitation department 15 days later, his anticoagulation drug was changed to rivaroxaban (20 mg, orally, once daily). Three months after discharge, the patient returned for another CTPA examination, which showed that there was no embolism in the main pulmonary artery or its branches (Fig. 3C1–5). Therefore, to prevent thrombosis, the dose of rivaroxaban was reduced to 10 mg once a day. The full timeline of hospitalization and clinical treatments is shown in Figure 5. Half a year later, the patient came to our hospital for review, and the CTPA result showed that no thrombus was seen, and the D-dimer was also in the normal range, so he switched to aspirin (100 mg, orally, once daily) treatment until now. At present, the patient general condition is stable and he can tolerate light physical activities.

## 3. Discussion

This study reports a 63-year-old male with acute massive brainstem infarction and high-risk PE. Due to thrombolysis contraindication, multidisciplinary pulmonary thrombectomy with local urokinase thrombolysis achieved rapid hemodynamic stabilization. Stepwise anticoagulation led to complete thrombus resolution on 3-month CTPA and neurological recovery. The case validates mechanical thrombectomy as pivotal for high-risk PE when thrombolysis is contraindicated, highlighting multidisciplinary collaboration and precision anticoagulation in managing complex dual pathologies.

Cerebral infarction and PE are the second and third fatal acute cardiovascular diseases after myocardial infarction.^[5]^ To the best of our knowledge, the acquired risk factors for pulmonary thrombosis include aging, hospitalization, surgery, tumors, limb immobilization, infection, and others.^[6–8]^ Most stroke patients have multiple risk factors for venous thrombosis, among which inactivity is particularly important.^[3]^ A fatal PE usually occurs 2 weeks after stroke and reaches its peak at the end of 2 weeks.^[9]^ Studies have shown that without prompt intervention, about 30% of such patients develop pulmonary emboli, and the incidence of severe hemiplegia can be as high as 70%.^[3]^ The patient in the case presented here was elderly; he was bedridden after stroke and had limited left-leg mobility; all these factors contributed to his pulmonary thrombosis. The PE appeared in the second week after stroke, which is consistent with the peak time of onset reported in the literature.

The clinical symptoms of PE are often atypical, leading to 13% to 25% of early deaths after stroke, and PE is also the most common cause of death in the early stages of stroke.^[3]^ Therefore, the early prevention and identification of PE are crucial. For individuals at high risk for venous thrombosis – after fully evaluating the bleeding risk of anticoagulant therapy – appropriate measures should be taken as early as possible to prevent the development of thrombosis.

In terms of treatment, patients with acute cerebral infarction combined with high-risk PE have complex conditions. How to balance anticoagulant therapy with secondary intracranial hemorrhage is a particular challenge. At present, the guidelines do not explicitly propose treatment strategies for such patients; thus, the approach has been based mainly on case reports.

Pavesi et al^[10]^ reported on a patient with high-risk PE complicated by cerebral infarction, who, after receiving thrombolytic therapy, received unfractionated heparin anticoagulation for 72 hours and was then started on oral warfarin therapy for 3 months. He underwent surgical closure of the foramen ovale 3 months later. No bleeding events occurred during this period, and the prognosis was good. Similarly, Fujino et al^[11]^ reported on a patient with cerebral infarction complicated by PE, who was treated with continuous intravenous unfractionated heparin until the condition stabilized; he was then switched to oral anticoagulant therapy with edoxaban, which finally led to a good result. However, Nam et al^[12]^ reported on another patient with a high-risk PE, who, after pulmonary artery thrombectomy, developed right hemiplegia and was unable to recover neurologic function over the long term. Eventually, he was transferred to a hospital specializing in conservative treatment. Our case was complicated by a pulmonary artery embolism accompanied by a large area of brainstem infarction, a condition known to have a high mortality rate, and the risk of bleeding during thrombolytic therapy in the acute stage of brainstem infarction is extremely high. Once such bleeding occurs, the consequences are unimaginable. According to the PE guidelines, unfractionated heparin is recommended as the first choice for patients with acute high-risk PE who require initial anticoagulation prior to thrombolytic therapy. In our case, therefore, anticoagulation with unfractionated heparin was given first, and once initial anticoagulation had failed, pulmonary artery thrombectomy was performed immediately.

From the perspective of cerebral infarction, some scholars hold that low-molecular-weight heparin is not useful for preventing deep veinous thrombosis because it increases the risk of intracranial hemorrhage.^[13]^ Others suggest that in patients with cerebral infarction, the use of unfractionated heparin after thrombolysis versus simple thrombolysis would not increase the risk of bleeding.^[14]^ From the perspective of PE, they hold that if there is no contraindication to thrombolysis, systemic thrombolysis is the preferred strategy for patients with high-risk PE. We, however, maintain that surgical thrombectomy or interventional thrombectomy should be the first choice.^[15]^ The advantages and disadvantages of these 3 approaches are shown in Table 2. Our patient with a high-risk PE was in the acute stage of a massive brainstem infarction, thrombolysis was contraindicated, and his clinical symptoms still deteriorated under anticoagulation therapy; thus, we realized that it would be useless to rely solely on anticoagulant treatment. Patients with a hemodynamically unstable PE in whom thrombolysis is contraindicated should immediately undergo pulmonary artery thrombectomy.^[16]^ This is a mechanical interventional procedure that, under the guidance of imaging, uses endovascular devices to remove a blood clot or thrombus.^[17]^ Thrombectomy is commonly used in acute ischemic stroke, acute myocardial infarction, and PE.^[18]^ Therefore, after discussion with a multidisciplinary team, we decided to perform pulmonary artery thrombectomy in our case. After the procedure, urokinase was administered via a catheter for selective thrombolysis, followed by intravenous maintenance anticoagulation with unfractionated heparin. This therapeutic schedule not only avoids the high risk of bleeding caused by intravenous thrombolysis in the acute stage of stroke but also greatly reduces the load of thrombi in the patient pulmonary arteries, thereby buying the time for subsequent anticoagulation and ultimately improving the patient prognosis. Of course, this study is a single-center case report with a sample size limited to one patient. The generalizability of the findings and conclusions is inherently constrained. Due to individual variability and the complexity of comorbid conditions, the efficacy and safety of the described therapeutic strategy require validation through larger cohort studies.

**Table 2:** Advantages and disadvantages of reperfusion regimens for high-risk pulmonary embolism of this patient.

Reperfusion regimens	Advantages	Disadvantages
Systemic thrombolysis	Noninvasive	High risk of bleeding
	Low-cost	
	Highly efficient in vascular recanalization	
Percutaneous catheter-directed treatment	Minimally invasive	Higher cost
	Highly efficient in vascular recanalization	
	Low risk of bleeding	
Surgical embolectomy	Highly efficient in vascular recanalization	Invasive; high cost
	Low risk of bleeding	Many complications: vascular rupture, vascular dissection, vascular wall damage, etc

## 4. Conclusion

In summary, early prevention and identification of PE are crucial in patients with acute stroke. In treating PE, the pros and cons should be weighed based on the severity of the patient condition, and personalized treatment strategies should be developed. Once a high-risk PE has developed in a patient with acute stroke, thrombolytic therapy is an absolute contraindication. When intravenous anticoagulants cannot improve hemodynamics, pulmonary artery thrombectomy is required.

## Acknowledgments

The authors would like to thank the nurses and residents of the emergency department and the neurology ward of the Affiliated Dongyang Hospital of Wenzhou Medical University, Dongyang, China, who were involved in the management of the patient. We thank LetPub (www.letpub.com) for its linguistic assistance during the preparation of this manuscript.

## Author contributions

**Data curation:** Qiong-Fang Yang.

**Conceptualization:** Cai-Min Shu.

**Investigation:** Qiong-Fang Yang.

**Supervision:** Cai-Min Shu.

**Writing – original draft:** Qiong-Fang Yang.

**Writing – review & editing:** Cai-Min Shu.
